# Patient costs for drug-resistant TB diagnosis and pre-treatment evaluation in North India

**DOI:** 10.5588/pha.24.0018

**Published:** 2024-09-01

**Authors:** S. Das, R. Kumar, A. Krishnan, S. Kant, A. Mohan

**Affiliations:** ^1^ Centre for Community Medicine, and; ^2^ Department of Pulmonary, Critical Care and Sleep Medicine, All India Institute of Medical Sciences, New Delhi.

**Keywords:** TB, cost, catastrophic, patient cost, India

## Abstract

**INTRODUCTION:**

India's National TB Elimination Programme (NTEP) aims to eliminate TB-related catastrophic expenditure by offering free diagnosis and treatment. However, 3.9% of TB patients have drug-resistant TB (DR-TB) and are facing higher costs.

**OBJECTIVE:**

To assess DR-TB patients' diagnosis and pre-treatment evaluation costs, catastrophic cost incidence, and its relation to patient characteristics.

**METHODS:**

The study included DR-TB patients from three District Drug-Resistant TB Centres in Delhi and Faridabad (October 2021–June 2022). Socio-economic and clinical characteristics and direct medical and non-medical costs from drug susceptibility testing eligibility to the start of DR-TB treatment were collected using patient interviews and records. Indirect costs were calculated via the human capital approach, defining catastrophic costs as expenses over 20% of household annual income. Multivariable regression was used to estimate the effects of patient characteristics on catastrophic costs.

**RESULTS:**

Of 158 patients, 37.3% were aged 19–30 years, and 55.7% were women. Median total cost was USD326.6 (IQR 132.7–666.7), with 48.2% for diagnosis and 66.0% indirect. 32% faced catastrophic costs, with manual labourers at higher risk (adjusted OR 4.4).

**CONCLUSION:**

Despite free diagnosis and treatment, a significant portion of DR-TB households in India incur catastrophic costs, mainly from indirect expenses, indicating a need for targeted policy and programme interventions.

## Abstract (fr)

**INTRODUCTION:**

Le Programme national Indien d'élimination de la TB (NTEP) a pour objectif de réduire les dépenses catastrophiques liées à la TB en offrant un diagnostic et un traitement gratuits. Cependant, 3,9% des patients atteints de TB présentent une TB résistante aux médicaments (DR-TB) et doivent faire face à des coûts plus élevés.

**OBJECTIF:**

Évaluer les coûts de diagnostic et d'évaluation pré-thérapeutique chez les patients atteints de DR-TB, ainsi que l'impact des coûts catastrophiques et leur corrélation avec les caractéristiques des patients.

**MÉTHODES:**

L'étude a porté sur les patients atteints de DR-TB provenant de trois Centres de lutte contre la TB résistante aux médicaments des districts de Delhi et de Faridabad, Inde (octobre 2021–juin 2022). Les données relatives aux caractéristiques socio-économiques et cliniques, ainsi qu'aux coûts directs médicaux et non médicaux, ont été collectées lors de l'évaluation de l'éligibilité à l'antibiogramme au début du traitement de la DR-TB, à travers des entretiens avec les patients et l'analyse des dossiers. Les coûts indirects ont été évalués en utilisant l'approche du capital humain, définissant les coûts catastrophiques comme dépassant 20 % du revenu annuel du ménage. Une régression multivariable a été réalisée pour estimer l'impact des caractéristiques des patients sur les coûts catastrophiques.

**RÉSULTATS:**

Sur un échantillon de 158 patients, 37,3% avaient entre 19 et 30 ans et 55,7% étaient des femmes. Le coût médian total s'élevait à 326,6 USD (IQR 132,7–666,7), dont 48,2% pour le diagnostic et 66,0% pour les coûts indirects. En outre, 32% des patients ont été confrontés à des coûts catastrophiques, les travailleurs manuels étant les plus touchés (OR ajusté 4,4).

**CONCLUSION:**

Bien que le diagnostic et le traitement soient gratuits, de nombreux ménages indiens touchés par la DR-TB doivent faire face à des coûts élevés, en particulier des dépenses indirectes, soulignant ainsi le besoin d'interventions politiques et programmatiques ciblées.

TB is a major global health problem, with India being the country with the highest burden of TB. According to the Global TB Report 2023, there were an estimated 10.6 million cases of TB worldwide in 2022, with 1.3 million deaths.^1^ Drug-resistant TB (DR-TB) is a form of TB that is resistant to one or more of the standard drugs used to treat the disease. DR-TB is a major public health threat, particularly in low- and middle-income countries, where TB is most prevalent. The burden of drug-resistant TB (DR-TB) increased by 3% between 2020 and 2021, with 450,000 new cases of rifampicin-resistant TB (RR-TB) in 2021. Globally, 3.3% of new TB patients and 17% of TB patients with a history of preceding medication for TB were identified with multidrug-resistant TB (MDR-TB), which is defined as TB which is resistant to both isoniazid (H, INH) and rifampicin (R, RIF) without resistance to any other first-line anti-TB drugs.^2^ The India TB Report 2023 states that the estimated proportion of MDR/RIF-resistant TB cases among new patients was 3.6% and 18% among previously treated patients.^3^

The End TB Strategy of the WHO has set a target to eliminate TB by the year 2035.^4^ Sustainable Development Goals (SDGs) have a similar target of eliminating TB by 2030.^5^ In line with these global commitments, the National Strategic Plan for Tuberculosis Elimination (NTEP) in India has set a target to eliminate TB by 2025.^6^ Elimination of catastrophic cost is one of the key indicators of TB elimination. However, there was no in-built mechanism in the NTEP to monitor the patients’ cost of care. The WHO defines catastrophic cost as the total cost of TB care (including direct and indirect costs) exceeding 20% of the annual income of the household.^7^ Poverty is both a risk factor and a consequence of TB, and it disproportionately affects households with low socio-economic status, causing a financial burden on them. This relationship between poverty and TB could result in detrimental outcomes like delayed care-seeking, which potentially increases the costs incurred from direct costs (for consultation, medications, investigations, travel, food, accommodation and hospitalisation), as well as from indirect costs (due to loss of productivity, resulting in loss of pay and job losses).

NTEP in India commits to providing free diagnosis and treatment facilities to patients with TB. Very few studies explored the costs incurred when seeking care for drug-resistant TB. Despite the provision of free diagnosis and treatment services under NTEP, one of the studies reported a substantial cost (direct and indirect form) incurred by patients with DR-TB who use these services.^8^ Hence, this study was conducted to generate more evidence regarding the cost incurred in diagnosis and pre-treatment evaluation among patients enrolled under NTEP in north India.

## METHODS

A community-based cross-sectional study was conducted among DR-TB patients between October 2021 and June 2022. This study was conducted at three district DR-TB centres (DDRTBCs) – one in Faridabad in the Haryana State in North India, and two (New Delhi Municipal Council Chest Clinic and Deen Dayal Upadhyay Hospital [DDUH] Chest Clinic, New Delhi, India) in the National Capital Territory (NCT) of Delhi. Approximately 15 patients with DR-TB are enrolled every month in DDRTBC Faridabad, while NDMC Chest Clinic received approximately 10 patients and DDUH Chest Clinic received approximately 15 patients. All cases of DR-TB registered under NTEP during the study period at the selected DDRTBCs were approached for inclusion. Patients who were on retreatment for DR-TB, those who were too sick to cooperate or unable to comprehend the questionnaire or who died or were lost to follow-up before the administration of the questionnaire were excluded from the study.

A structured questionnaire adapted from the “Tool to estimate patients’ cost” developed by KNCV (Royal Dutch Central Association), WHO and Japan Anti-TB Association was used in the study.^9^ Information on direct medical costs, which included costs incurred on consultation, investigations, medications and hospitalisation charges, and on direct non-medical costs, which included transportation, food and lodging charges, were taken through the administration of a questionnaire on patients and/or caregivers and medical receipts of the patient wherever available. Indirect cost was primarily calculated through the human capital method with equity correction.^7,10^ Information on work absenteeism was obtained from patients and caregivers. In the human capital method, the pre-TB monthly income of the patient and caregiver was converted into daily wage and was multiplied by the absenteeism days to calculate the indirect cost. For those adults who were not earning (e.g., students and homemakers), equity correction was added by using the minimum daily wage for unskilled labour for the state of Haryana (USD4.2) and NCT of Delhi (USD7.5), depending on the patients’ residence.^11,12^ For sensitivity analysis, two other methods were also used and compared: 1) human capital method without equity correction; 2) output method (calculated as a difference between the family income before the onset of TB and after treatment initiation).^7^

Analysis was done using STATA v17.0 (StataCorp, College Station, TX, USA). Mean (standard deviation [SD]) and median costs (interquartile range [IQR]) incurred in the diagnosis and the pre-treatment evaluation are presented. Direct costs incurred per visit during diagnosis of DR-TB were calculated separately for public and private health facilities by dividing the total costs of the total number of visits at both facilities. Households experiencing catastrophic cost of TB were presented as a proportion with a 95% confidence interval. All costs were reported as United States dollars (USD) according to the Reserve Bank of India’s (RBI’s) USD and Indian rupee (INR) exchange rate, which is 1USD = INR83.05.^13^ Sociodemographic and clinical characteristics of the study patients were reported as proportions or means/medians (with SD/IQR) where appropriate. The wealth index was calculated using a principal component analysis of socio-economic characteristics (occupation, ownership of assets, and housing characteristics). Logistic regression was done to look for determinants of catastrophic costs. Multivariable regression models were used to estimate the association of socio-demographic and disease characteristics with catastrophic costs. Variables for which the *P* < 0.2 were included in multivariable regression. The level of significance taken was 5%.

Before starting the study, ethical clearance was obtained from the All India Institutes of Medical Sciences Ethics Committee for Post-Graduation Research, New Delhi, India (IECPG–748/23.12.2021). The entire study procedure was explained to the participants and informed written consent was obtained from them. During the course of the study, patients were provided appropriate medical advice by a physician or were referred to higher centres for the management of complications. The digital records were kept password-protected and had limited access. Confidentiality about the identity of the participant was maintained throughout the study.

## RESULTS

Between 1 October 2021 and 30 June 2022, 192 patients were treated for DR-TB in the three selected DDRTBCs. Among the eligible patients, 17 could not be contacted, 5 died, 6 were lost to follow-up, and 6 refused to participate. Finally, 158 patients with DR-TB were enrolled in this study.

Most of the patients belonged to the 19–30 years age group (37.3%), were female (55.7%), residing in an urban area (85.4%), had completed 6–12 years of education (53.8%) and were unemployed (60.8%). The median annual family income was USD2456.4 (IQR 1733.9–4334.7). The mean age of all participants was 28.9 years (SD ±13.7), while the mean years of completed education was 8 years (SD ±4). Four-fifths of the patients had pulmonary TB, three-fourths of the patients had multidrug MDR/RR-TB and 58% of the patients were enrolled in a longer oral MDR/extensively drug-resistant TB regimen. Two-thirds of the patients had no past episode of TB. Forty-five (28.5%) of the patients had one episode of TB in the past, and six (3.8%) had more than one episode. Before treatment initiation, 63 (39.9%) of patients had their sputum microscopy for acid-fast bacilli (AFB) done, 130 (82.3%) had their cartridge-based nucleic acid amplification test (CBNAAT) done, 35 (22.2%) had their line probe assay done, and only two (1.3%) patients had their culture done (Table 1).

**TABLE 1.** Characteristics of patients with DR-TB enrolled from Delhi and Faridabad, India.

Variable	Category (*n* = 158)	*n* (%)
Age group, years	≤18	42 (26.6)
	19–30	59 (37.3)
	31–50	43 (27.2)
	>50	14 (8.9)
Sex	Male	70 (44.3)
Residence	Rural	23 (14.6)
	Urban	135 (85.4)
Region	Delhi	79 (50)
	Faridabad	79 (50)
Religion	Hindu	137 (86.7)
	Other	21 (13.3)
Years of education	≤5 years	44 (27.9)
	6–12 years	85 (53.8)
	>12 years	29 (18.3)
Occupation	Formal employees, and sales workers	24 (15.1)
	Manual workers and elementary occupations	38 (24.1)
	Unemployed (including students and housewives)	96 (60.8)
Monthly family income (pre-TB), USD, median [IQR]		204.4 [144.3–360.7]
Monthly personal income (pre-TB), USD, median [IQR]		0 [0–120.3]
Ever use of smoking tobacco		29 (18.3)
Ever use of smokeless tobacco		22 (13.9)
Ever use of alcohol		29 (18.3)
Wealth Index (in terciles)	I (poorest)	53 (33.5)
	II	53 (33.5)
	III (richest)	52 (33.0)
Site of TB	Pulmonary	133 (84.2)
	Extrapulmonary	25 (15.8)
Type of DR-TB case	INH mono/poly DR-TB	19 (12.0)
	MDR/RR-TB	119 (75.3)
	Pre-XDR-TB	14 (8.9)
	XDR-TB	6 (3.8)
Treatment regimen	INH mono/poly regimen	17 (10.8)
	Shorter oral BDQ-containing MDR/RR-TB regimen	46 (29.1)
	Longer oral M/XDR-TB regimen	92 (58.2)
	BPaL regimen	3 (1.9)
History of TB	New (no past episode of TB)	107 (67.7)
	Retreatment (≥1 past episodes of TB)	51 (32.3)
Episodes of TB in past, *n*	0	107 (67.7)
	1	45 (28.5)
	≥2	6 (3.8)
Diagnostic tests done before DR-TB treatment initiation		
AFB microscopy		63 (39.9)
CBNAAT		130 (82.3)
Line-probe assay		35 (22.2)
Culture		2 (1.3)

DR-TB = drug-resistant TB; USD = US dollar; IQR = interquartile range; INH = isoniazid; MDR/RR-TB = multidrug- /rifampicin-resistant TB; XDR-TB = extensively drug-resistant TB; BDQ = bedaquiline; BPaL = bedaquiline + pretomanid + linezolid; AFB = acid-fast bacilli; CBNAAT = cartridge-based nucleic acid amplification test.

The median total cost incurred on diagnosis of DR-TB and pre-treatment evaluation was USD326.6 (IQR 132.7–667.9). The median total direct medical cost was USD39.0 (IQR 7.7–138.3), accounting for 24.8% of the total cost. The median total direct non-medical cost was USD28.1 (IQR 12.0–58.9), accounting for 9.2% of the total cost. The median total indirect cost was USD124.2 (IQR 40.1–446.1), accounting for 66.0% of the total cost. The cost of diagnosis of DR-TB (87.5% of total cost) was higher than the cost of pre-treatment evaluation. Most of u`the direct costs incurred during diagnosis of DR-TB were on medications (10%) followed by blood investigations (7.8%). During the pre-treatment evaluation, most of the direct costs incurred were on travel (18.2%) followed by blood investigations (8.4%) (Figure, Table 2 and Supplementary Table S1). During the diagnosis of DR-TB, direct medical costs of each visit to a private health facility were higher by a mean difference of USD24.7 compared to one such visit to a public health facility, with the highest contributor being the medication costs. We also found that during the diagnosis of DR-TB, direct non-medical costs of every visit to a public health facility are higher by a mean difference of USD0.6 than one visit to a private health facility, with the highest contributor being the travel costs (Supplementary Table S2). DR-TB has resulted in patients stopping to work and loss of jobs. Of 62 patients employed, 43 (69.4%) of them had stopped working in their professions, and 14 (22.5%) had lost their jobs (Table 3). The proportion of patients experiencing catastrophic costs was 31.7% (95% confidence interval [CI] 24.5–39.5). In the sensitivity analysis, depending upon the approach used for the calculation of indirect cost, the proportion of households facing catastrophic cost was 9.5% (95% CI 5.4–15.2) (output method) and 22.8% (95% CI 16.5–30.1) (human capital method). Factors potentially associated (*P* < 0.2) with the catastrophic cost in the bivariable analysis were age, gender, religion, wealth index and occupation. In the multivariable analysis, patients employed as manual workers or in elementary occupations were more likely (adjusted odds ratio [aOR] 4.4, 95% CI 1.3–15.7) to experience catastrophic expenditure (Table 4).

**FIGURE.**
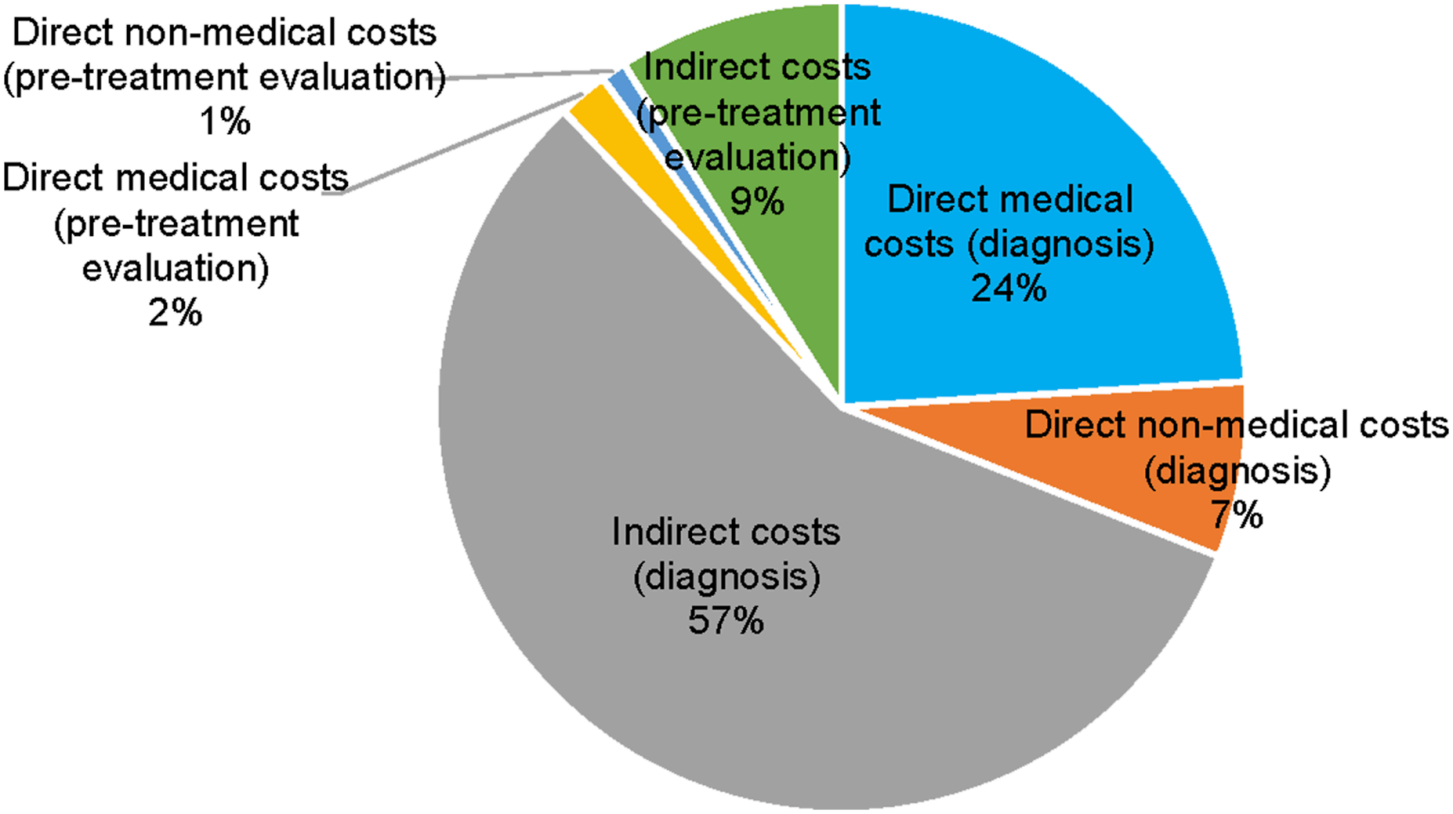
Total cost of diagnosis and treatment initiation for DR-TB among patients enrolled from Delhi and Faridabad, India. DR-TB = drug-resistant TB.

**TABLE 2.** Costs incurred in diagnosis and pre-treatment evaluation among patients with DR-TB enrolled from Delhi and Faridabad, India.

Type of cost (*n* = 158)	Cost of diagnosis of DR-TB	Cost of pre-treatment evaluation	Total cost
	Median [IQR]	Median [IQR]	Median [IQR]
Direct medical cost, USD	35.13 [1.39–138.29]	0 [0–6.01]	39.03 [7.70–138.29]
Consultation cost	1.27 [0–10.91]	0 [0–0.06]	1.37 [0–10.91]
Medication cost	7.22 [0–30.06]	0 [0–0]	7.22 [0–30.06]
Test costs (blood tests, sputum test)	6.01 [0–31.27]	0 [0–3.61]	12.03 [0–40.89]
X ray/CT scan cost	0.60 [0–12.03]	0 [0–0]	3.01 [0–12.03]
MRI/FNAC/biopsy cost	0 [0–0]	—	0 [0–0]
Hospitalisation cost	0 [0–0]	—	0 [0–0]
Direct non-medical cost, USD	21.54 [8.86–42.35]	6.41 [2.72–13.71]	28.14 [12.03–58.92]
Travel cost	21.31 [7.68–41.91]	6.61 [2.87–13.87]	28.14 [11.06–57.22]
Food cost	0 [0–0]	0 [0–0]	0 [0–0]
Accommodation cost	0 [0–0]	0 [0–0]	0 [0–0]
Indirect cost, USD	107.12 [34.68–385.29]	17.15 [5.62–60.64]	124.27 [40.08–446.13]
Total cost, USD	272.41 [100.15–575.90]	44.73 [16.08–83.05]	326.64 [132.73–666.74]

DR-TB = drug-resistant TB; USD = US dollar; IQR = interquartile range; CT = computed tomography; MRI = magnetic resonance imaging; FNAC = fine-needle aspiration cytology.

**TABLE 3.** Impact on employment of patients with drug-resistant TB enrolled from Delhi and Faridabad, India.

Occupation	Total	Pre-TB personal income (USD)	People who stopped working	Time absent from work Days	Job loss
	*n*	Median [IQR]	*n* (%)	Median [IQR]	*n* (%)
Manual workers and elementary occupations	38	132.5 [96.3–180.6]	26 (68.5)	45 [0–120]	9 (23.7)
Formal employees and sales workers	24	144.5 [114.4–301.0]	17 (70.8)	45 [0–130]	5 (20.8)
Total	62		43 (69.4)		14 (22.5)

USD = US dollar; IQR = interquartile range.

**TABLE 4.** Determinants of catastrophic costs among drug-resistant TB patients (plus caregivers) in the three selected DDRTBCs.

Variable		Total	Catastrophic cost	Bivariable analysis	Multivariable analysis
		(*n*)	*n* (%)	cOR (95% CI)	aOR (95% CI)
Age group, years	>50	14	2 (14.3)	Reference	Reference
	≤18	42	19 (45.2)	5.0 (1.0–24.9)*	2.4 (0.3–16.7)
	19–30	59	14 (23.7)	1.9 (0.4–9.4)	1.6 (0.3–9.6)
	31–50	43	15 (34.9)	3.2 (0.6–16.3)*	2.6 (0.5–14.3)
Age (for every 1-year increase)				1.0 (0.9 – 1.0)	Not done
Sex	Female	88	20 (22.7)	Reference	Reference
	Male	70	30 (42.9)	2.6 (1.3–5.1)*	1.1 (0.4–3.3)
Residence	Rural	23	7 (30.4)	Reference	—
	Urban	135	43 (31.9)	1.1 (0.4–2.8)	—
Region	Delhi	79	22 (27.9)	Reference	—
	Faridabad	79	28 (35.4)	1.4 (0.7–2.8)	—
Religion	Hindu	137	40 (29.2)	Reference	Reference
	Others	21	10 (47.6)	2.2 (0.9–5.6)*	1.7 (0.6–5.1)
Wealth Index	I (poorest)	53	23 (43.4)	Reference	Reference
	II	53	13 (24.5)	0.4 (0.1–0.9)*	0.7 (0.2–1.8)
	III (richest)	52	14 (26.9)	0.5 (0.2–1.1)*	1.0 (0.4–2.5)
Years of education	≤5 years	44	11 (25.0)	Reference	—
	6–12 years	85	30 (35.3)	1.6 (0.7–3.7)	—
	>12 years	29	9 (31.0)	1.4 (0.5–3.8)	—
Years of education (for every 1-year increase)				1.0 (0.9 – 1.1)	—
Site of TB	Pulmonary	133	42 (31.6)	Reference	—
	Extrapulmonary	25	8 (32.0)	1.0 (0.4–2.5)	—
History of smoking	Yes	29	11 (37.9)	Reference	—
	No	129	39 (30.2)	0.7 (0.3–1.6)	—
History of smokeless tobacco	Yes	22	7 (31.8)	Reference	—
	No	136	43 (31.6)	1.0 (0.4–2.6)	—
History of alcohol	Yes	129	38 (29.5)	Reference	—
	No	29	12 (41.4)	1.7 (0.7–3.9)	—
First facility visited	Government	78	22 (28.2)	Reference	—
	Private	80	28 (35.0)	1.4 (0.7–2.7)	—
Occupation	Unemployed (including students and housewives)	96	26 (27.1)	Reference	Reference
	Formal employees and sales workers	24	9 (37.5)	1.6 (0.6–4.1)	3.7 (1.0–14.4)
	Manual workers and elementary occupations	38	15 (39.5)	1.8 (0.8–3.9)*	4.4 (1.3–15.7)^†^

* *P* < 0.2.

† *P* < 0.05.

DDRTBC = district DR-TB centre; cOR = crude odds ratio; aOR = adjusted OR.

## DISCUSSION

We found significant costs involved in diagnosing and initiating treatment for drug-resistant TB among patients enrolled in the NTEP. Direct medical cost accounted for 24.8%, direct non-medical cost accounted for 9.2%, and indirect cost accounted for 66.0% of the total cost. Around one-third of patients experienced catastrophic costs.

We found that the median total cost of diagnosis and treatment initiation for DR-TB was USD326.64, which is higher than a similar study done in India (median total cost = USD192.8 following yearly inflation from 2016 to 2023).^8^ It is lower than a study done in Cambodia (median total cost = USD470.33 after yearly inflation from 2008 to 2023) and higher than a study done in Ethiopia (median total cost = USD109.72 after yearly inflation from 2013 to 2023).^14,15^ We also found that the indirect costs constituted 66.0% of the total cost, indicating that greater costs are incurred due to loss of productivity and income potential because of the disease. This is comparable to the study by Collins et al. in which indirect costs comprised 55.4% of the total cost.^15^ We found that during the diagnosis of DR-TB, direct medical costs of each visit to a private health facility were higher by a mean difference of USD24.7 compared to one such visit to a public health facility, with the highest contributor being the medication costs. We also found that during the diagnosis of DR-TB, direct non-medical costs of every visit to a public health facility are higher by a mean difference of USD0.6 than one visit to a private health facility, with the highest contributor being the travel costs. It is speculated that private health facilities influence the preference for public health facilities due to the lesser access and greater distance from patients’ homes.

Overall, 32% of DR-TB patients experienced catastrophic costs (total cost >20% of household income)^7^ from initial diagnosis of TB to initiation of treatment for DR-TB. Catastrophic costs were determined by employment (manual workers had greater odds of incurring higher catastrophic costs than other professions). None of the studies conducted in the past had reported catastrophic expenditure exclusively in the diagnostic and pre-treatment evaluation pathways of the patients.^8,14–18^ Unfavourable employment, commitment of patients to their families for their upkeep, long distances of DR-TB centres and testing facilities from their homes, and high costs of pre-treatment initiation investigations could be factors leading to higher costs incurred by patients in the diagnostic pathway, as reported by similar studies.^19–27^

Our study had some strengths and limitations. Unlike other studies, costs incurred in diagnosis and treatment initiation were presented in our study in a detailed disaggregated format according to WHO tools for cost analysis in TB, thereby helping in ease of interpretation. One of our limitations was that the sample size was inadequate to assess the association between catastrophic costs and the characteristics of the participants. Therefore, we might have missed the patient’s characteristics even though they might have been associated. Also, patients were not recruited directly from the community. Hence, presumptive DR-TB patients who accessed care from private facilities were missed out. Therefore, we might have failed to identify the costs incurred in diagnosis and treatment initiation among private facility attendees.

Building on the results, we recommend increasing the number of public health facilities equipped with rapid TB diagnostics to facilitate patient access. We also recommend changing the availability times of the public health facilities for consultation and investigation so that hindrances in access to care due to employment are reduced.

## CONCLUSION

Officially, DR-TB diagnostic and treatment services are provided to patients in India at no cost; yet, patients bear substantial expenses and frequently experience job loss due to the stigma of TB, resulting in severe income decreases. If the patient is the family's primary provider, additional expenses and missed income are typically disastrous. A significant financial burden may prevent individuals from receiving a diagnosis, beginning treatment, or stopping it altogether, which might prolong the illness's spread to other people and raise medication resistance. The results of this investigation point to the necessity of laws and procedures that lessen the financial strain on DR-TB patients and their families.

## Supplementary Material


