# Supplementary material for: Patient costs for drug-resistant TB diagnosis and pre-treatment evaluation in North India
Source: Public Health Action. 2024 Sep 1;14(3):129–34. doi: 10.5588/pha.24.0018 (PMC11373743; doi:10.5588/pha.24.0018)
Supplement: Supplementary file 1 [file iutld_pha_24-0018_supplementarydata1.pdf]

SUPPLEMENTARY TEXT

Supplementary Table 1: Mean costs incurred in diagnosis and pre-treatment evaluation among patients with drug-resistant tuberculosis enrolled from Delhi and Faridabad, India

Type of cost (in USD) (N=158)	Cost of diagnosis of DR-TB, in Mean (SD)	Cost of pre-treatment evaluation, in Mean (SD)	Total cost, in Mean (SD)	% of mean total cost
**Direct Medical Cost**	**122.06 (228.41)**	**6.37 (17.74)**	**128.43 (229.19)**	**24.8**
Consultation cost	13.71 (32.66)	0.02 (0.06)	13.73 (32.66)	2.7
Medication cost	45.40 (97.85)	0.29 (1.97)	45.68 (97.99)	8.8
Test costs (blood tests, sputum test)	35.35 (83.91)	5.42 (16.94)	40.78 (86.17)	7.9
X ray / CT scan cost	13.35 (34.06)	0.64 (2.41)	13.99 (34.02)	2.7
MRI / FNAC / Biopsy cost	6.79 (24.78)	-	6.79 (24.78)	1.3
Hospitalization cost	7.70 (34.22)	-	7.70 (34.22)	1.5
**Direct Non-medical cost**	**35.39 (65.51)**	**11.89 (16.91)**	**47.28 (74.97)**	**9.2**
Travel cost	34.20 (61.61)	11.87 (17.16)	46.03 (71.56)	8.9
Food cost	0.96 (6.22)	0.05 (0.39)	1.01 (6.64)	0.2
Accommodation cost	0.23 (2.83)	0.01 (0.24)	0.24 (3.07)	0.1
**Indirect cost**	**294.81 (426.24)**	**46.51 (66.72)**	**341.32 (492.95)**	**66.0**
**Total cost**	**452.26 (504.69)**	**64.78 (69.82)**	**517.04 (565.06)**	**100**

Supplementary Table 2: Direct costs (in USD) incurred in diagnosis and pre-treatment evaluation per visit among patients with drug-resistant tuberculosis enrolled from Delhi and Faridabad, India

Type of cost during diagnosis of DR-TB (in USD) (N=158)	On every one visit in public health facilities		On every one visit in private health facilities	
	Mean (SD)	Median (IQR)	Mean (SD)	Median (IQR)
**Direct Medical Cost**	**7.59 (25.43)**	**0.04 (0 – 6.01)**	**31.93 (140.17)**	**5.41 (0 – 26.46)**
Consultation cost	0.02 (0.02)	0 (0 – 0.04)	3.73 (12.48)	0.61 (0 – 4.41)
Medication cost	0.70 (2.03)	0 (0 – 0)	10.62 (37.41)	0.96 (0 – 6.01)
Test costs (blood tests, sputum test)	5.33 (22.14)	0 (0 – 0.60)	9.74 (59.01)	0 (0 – 4.00)
X ray / CT scan cost	0.85 (2.55)	0 (0 – 0)	4.59 (26.68)	0 (0 – 1.80)
MRI / FNAC / Biopsy cost	0.69 (3.63)	0 (0 – 0)	1.13 (8.53)	0 (0 – 0)

Type of cost during diagnosis of DR-TB (in USD) (N=158)	On every one visit in public health facilities		On every one visit in private health facilities	
	Mean (SD)	Median (IQR)	Mean (SD)	Median (IQR)
Hospitalization cost	-	-	175 (1049)	0 (0 – 0)
**Direct Non-medical cost**	**2.09 (3.15)**	**1.29 (0.64 – 2.71)**	**1.86 (10.51)**	**0 (0 – 0.85)**
Travel cost	2.06 (3.15)	1.29 (0.60 – 2.41)	1.77 (10.00)	0 (0 – 0.85)
Food cost	0.04 (0.24)	0 (0 – 0)	0.07 (0.65)	0 (0 – 0)
Accommodation cost	-	-	0.02 (0.25)	0 (0 – 0)
**Total direct cost**	**9.67 (25.66)**	**2.84 (1.20 – 8.42)**	**33.78 (142.94)**	**6.18 (0 – 27.06)**
